# Development and validation of a self-report measure of epistemic trust

**DOI:** 10.1371/journal.pone.0250264

**Published:** 2021-04-16

**Authors:** Chloe Campbell, Michal Tanzer, Rob Saunders, Thomas Booker, Elizabeth Allison, Elizabeth Li, Claire O’Dowda, Patrick Luyten, Peter Fonagy

**Affiliations:** 1 Research Department of Clinical, Educational and Health Psychology, University College London, London, United Kingdom; 2 Anna Freud National Centre for Children and Families, London, United Kingdom; 3 Faculty of Psychology and Educational Sciences, KU Leuven, Leuven, Belgium; Medical University of Vienna, AUSTRIA

## Abstract

Epistemic trust (ET) refers to trust in communicated knowledge. This paper describes the development and validation of a new self-report questionnaire, the Epistemic Trust, Mistrust and Credulity Questionnaire (ETMCQ). We report on two studies (Study 1, n = 500; Study 2, n = 705) examining the psychometric properties of the ETMCQ and the relationship between EMTCQ scores (i.e., an individual’s epistemic stance) and exposure to adverse childhood experiences, mental health symptoms, attachment, mentalizing and general self-efficacy. The factor structure of the ETMCQ was examined using Exploratory and Confirmatory Factor Analyses, and its reliability and test-retest reliability were tested. Both studies yielded three correlated yet distinct factors–Trust, Mistrust and Credulity–and confirmed the reliability and validity of the ETMCQ. Preregistered hypotheses were confirmed and replicated across both studies. Main findings suggest intriguing links between the ETMCQ and developmental psychopathology constructs and are consistent with thinking on the role of epistemic stance in undermining adaptation and increasing the developmental risk of mental health problems. Mistrust and Credulity scores were associated with childhood adversity and higher scores on the global psychopathology severity index and both factors partially mediated the link between early adversity and mental health symptoms. Mistrust and Credulity were positively associated with difficulties in understanding mental states and insecure attachment styles. Post-hoc analysis identified that different attachment styles were associated with differences in epistemic stance. In addition, Trust was not associated with reduced levels of mental health symptoms and did not moderate the impact of childhood adversity–findings are congruent with the suggestion that the reduction of mistrust and credulity may be crucial common factors in promoting resilience and the effectiveness of psychotherapeutic interventions. This investigation and the ETMCQ provide an empirical measure of what until now has been largely a theoretical concept and open new avenues for future research.

## Introduction

Epistemic trust (ET) refers to trust in communication or communicated knowledge [1]. Specifically, it refers to the capacity of the individual to consider knowledge that is conveyed by others as significant, relevant to the self, and generalizable to other contexts [for other definitions see 2, 3]. Traditionally of interest primarily as a sociological and philosophical construct [4–6], it has recently been co-opted into theoretical explorations of the social-cognitive processes underpinning many expressions of psychopathology [7–9]. This thinking is supported by experimental data, in particular work by the developmental psychologists Gergely and Csibra, which suggests that interpersonal signals impact heavily on infants’ responses to the communication of cultural knowledge [10–13]. From a developmental perspective, infants appear sensitive to ‘ostensive cues’ from the communicator that signal the intention to transmit new and relevant information to them [10–12]. For example, 14–16 month-old infants who received cues alerting them to a communication personally relevant to them before observing an adult demonstrating an action on a target were more likely to imitate the action [14]. This pattern of results suggests that ostensive communication facilitates efficient learning of culturally shared knowledge as opposed to observational learning mechanisms alone [11]. It is argued that such ostensive cues trigger a pedagogic stance which opens a channel for communication of cultural knowledge, leading the recipient to encode what they are being told as relevant to them, generalizable and therefore should be remembered [15].

This framework has encouraged Fonagy and colleagues to go beyond the cognitive and social learning processes of infancy and to suggest that a position of ET allows individuals to acquire knowledge and accommodate new information in a way that supports resilient social functioning [7]. The concept of ET does not just concern an open stance to trust any given information or general trust [16], but rather a more complex process which depends on the individual’s (the addressee’s) ability to take into account the reliability of the source of information, the information’s relevance to the addressee, and the quality of the information itself [7]. The addressee expects the sender to communicate information that is the most relevant they could provide, considering his or her own beliefs and interests [17]. It is not advisable to accept everything we are told without discrimination: a self-protective capacity to critique information is necessary as some people might be unreliable informants, whether through ignorance or malevolent intentions. This position has been described as epistemic vigilance [1]. Addressees search for information that is relevant to them “by answering a question they had in mind, improving their knowledge on a certain topic, settling a doubt, confirming a suspicion, or correcting a mistaken impression” [18, p. 607] and exclude interpretations that seem irrelevant, overly obvious, or false. The addressee has the legitimate expectation that the informant or sender will communicate information consistent with his or her own mental states.

Fonagy et al. have suggested that ET is not only influenced by the immediate context (for example, if a speaker actually seems reliable), but it can also reflect a trait-like tendency to trust others, which underpins resilience to psychopathology. Critically, it is argued that the stance to trust socially transmitted information is rooted in developmental experiences such as attachment security, childhood adversity and the associated capacity to reflect on mental states (i.e., mentalizing). It could be one of the mechanisms mediating individual differences in social learning linked to histories of trauma and deprivation [e.g. 19, 20] and may explain some developmental vulnerabilities for psychopathology, in particular more severe forms of mental disorder [8, 9].

However, despite some intriguing initial evidence [21], empirical data on the relationship between ET and childhood adversity, attachment, psychological well-being and risk of psychopathology in adults is limited by the absence of a practical instrument to assess the construct. The objectives of the studies described here were therefore to develop and test a new scale to measure individuals’ epistemic stance, the Epistemic Trust, Mistrust and Credulity Questionnaire (ETMCQ), and to explore the relationship between the epistemic stance, mental health symptoms and developmental experiences.

### The epistemic stance: A three-factor construct

Inspired by the work of Grice [22] and Relevance Theory [17, 18], which maintains that human communication raises the specific expectation that speakers or senders intend to convey information that is relevant, we suggest three possible overlapping dimensions an individual may adopt in relation to social communication. The first is *epistemic trust*–an adaptive stance in relatively benign social circumstances, in which the individual is selectively and appropriately open to opportunities for social learning in the context of relationships, that can confer psychological resilience in the face of life challenges. *Epistemic mistrust* reflects a tendency to treat any source of information as unreliable or ill-intentioned, and to err on the side of rejecting or avoiding allowing oneself to be influenced by communication from others. *Epistemic credulity* pertains to a pronounced lack of vigilance and discrimination, signaling a general lack of clarity about one’s own position and resulting in vulnerability to misinformation and potential risk of exploitation. For convenience, in the text below we will refer to these positions as Trust, Mistrust and Credulity without the descriptor ‘epistemic’ but it should be remembered that these are positions in relation to (socially transmitted) knowledge and not interpersonal dispositions. Although these three dimensions may overlap, it is an empirical question whether they reflect a bipolar dimension (ranging from credulity to trust to mistrust), or whether these are three related, yet distinct, features of the epistemic stance. Clinically, it appears that these three dimensions may be relatively distinct. For instance, some individuals have both high levels of Credulity and Mistrust, as is typical of many individuals with antisocial and borderline personality disorder [23, 24]. Similarly, individuals with paranoid personality traits might be characterized by high levels of both Mistrust and Trust in others.

### The present study

In this paper, we report on two studies examining the psychometric properties of a new self-report measure of epistemic trust, the ETMCQ. We first describe the development of the items and then two investigations investigating its psychometric features. In the first study, 500 community individuals completed the ETMCQ online. We examined the factor structure of the ETMCQ using Exploratory Factor Analysis (EFA) and Confirmatory Factor Analysis (CFA), and its reliability using this sample. Test-retest reliability was analyzed using 441 (88%) of the original participants who agreed to complete the measure again. We expected a three-factor structure, with Trust, Mistrust and Credulity emerging as three correlated, yet distinct factors.

Study 2 was set up to replicate the factor structure of the ETMCQ in 705 community individuals. Given the theoretical developmental and clinical background of the ETMCQ, both studies also aimed to address the relations between the ETMCQ subscales and measures of exposure to adverse childhood experiences, mental health symptoms, attachment, mentalizing and general self-efficacy.

We preregistered our hypotheses and study design on OSF (https://osf.io/6p4ve). Our overall study objective, as pre-registered, was to establish how the epistemic stance might be linked to developmental experiences. In our pre-registration, we conceptualized Mistrust as an overarching construct encompassing Mistrust and Credulity in opposition to the more adaptive position on Trust. Our hypotheses were as follows:

1. Higher levels of Trust would be negatively associated with adverse childhood experiences and mental health symptoms and positively with general self-efficacy.
2. Both Mistrust and Credulity would be associated with more indices of adverse childhood experiences such as abuse, maltreatment and/or neglect, and with poor mentalization, elevated mental health symptoms and insecure attachments styles.
3. Given our hypothesis on the relationship between epistemic stance, developmental experiences and psychopathology, we predicted that the three ETMCQ factors would mediate the link between childhood adversity and mental health symptoms.
4. Trust would act as a resilience factor and buffer the negative impact of low self-efficacy and/or insecure attachment, such that individuals with higher levels of Trust and low self-efficacy or insecure attachment would report fewer mental health symptoms.

## Methods

### Development of the Epistemic Trust Questionnaire

In the initial development of the ETMCQ, we gathered a scale of 60 self-descriptor items covering three content categories: Epistemic Trust, Epistemic Mistrust and Epistemic Credulity. For example, a high Trust item is “I find information easier to trust and absorb when it comes from someone who knows me well”. An example of a high Mistrust item is “If you put too much faith in what people tell you, you are likely to get hurt”. A high Credulity item is “When I speak to different people, I find myself easily persuaded even if it is not what I believed before”. Responses were rated across a 7-point Likert scale anchored as “strongly disagree” (= 1) to “strongly agree” (= 7) and neither agree nor disagree in the center (= 4). High Epistemic Trust, Mistrust and Credulity was indicated on polar response items by either strong agreement (= 7) or strong disagreement (= 1) with the statement.

The sixty items were independently rated by six expert judges (clinicians and academics familiar with theories concerning ET) who indicated whether they thought each statement captured Trust, Mistrust or Credulity. In order to produce a questionnaire with six items per factor, 42 items were rejected on the basis of being badly worded, ambiguous, repetitious, or irrelevant. The expert judges’ decisions to retain items were based on their qualitative assessment of how well each item fitted the relevant factor. The eighteen remaining items were re-examined by the same expert judges and two additional expert judges. Following their feedback, items were refined to enhance clarity, relevance and readability; eighteen items were retained, six for each subscale. The items were then reviewed by ten non-experts (colleagues within the UCL psychoanalysis unit working on unrelated areas), who were asked to elaborate on their understanding of each item. Following their responses, the expert group re-examined the scale, adjusted some wording and replaced one item. This led to an 18-item version of the scale which was used in both studies.

### Participants and procedures

The ETMCQ was administered to 500 participants in Study 1 and 705 participants in Study 2, using the on-line survey platform Prolific (https://www.prolific.co), which includes an algorithm to recruit a representative sample that approximately matches the United Kingdom (UK) population distribution in terms of age (five brackets 18–27, 28–37, 38–47, 48–57, and 58+), sex (male and female) and ethnicity (five groups). Participants were aged 18 years or older, currently living in the UK, and proficient in written and spoken English, predominantly white with a mean age of 45.34 years in Study 1 and 44.34 years in Study 2 (see Table 1 for demographic characteristics). Participants received financial compensation (at a rate of £7.50 per hour). The study was approved by the University College London Research Ethics Committee (reference 14285/002). Questionnaires were designed in Qualtrics Online Sample (Qualtrics, Provo, UT). All participants were first asked to complete the demographic questionnaire (see Table 1), followed by a battery of questionnaires. The questionnaires were presented in random order, separately randomized for each participant. In order to establish test-retest reliability, we contacted all participants from Study 1 who had agreed to be re-contacted (N = 467), and asked them to fill out the ETMCQ again three weeks after the initial administration.

**Table 1:** Sample demographics for Study 1 (N = 500) and Study 2 (N = 705).

**Gender**					
	Female	Male	Non-Binary	Prefer not to say	
Study 1	255 (51%)	241 (48.2%)	2 (0.4%)	2 (0.4%)	
Study 2	363 (51%)	342 (49%)	0	0	
Age					
	**18–29**	**30–39**	**40–49**	**50–59**	**>60**
Study 1	100 (20%)	91 (18%)	87 (17%)	91 (18%)	131 (26%)
Study 2	172 (24%)	125 (18%)	93 (13%)	143 (20%)	172 (34%)
**Ethnicity**					
	White	Asian or Asian British	Black / African / Caribbean / Black British	Mixed / Multiple ethnic groups	Other/ Prefer not to say
Study 1	404 (81%)	48 (10%)	24 (5%)	15(3%)	9 (2%)
Study 2	584 (83%)	57 (8%)	32 (4.5%)	18(2.5%)	14(2%)
**Marital status**					
	Single	In a relationship/ civil/married	Separated/Divorced	Widowed	Prefer not to say
Study 1	107 (21%)	354 (71%)	31 (7%)	6 (2%)	2 (0.01%)
Study 2	191 (27%)	463 (66%)	37 (5%)	13 (2%)	1 (0.01%)
**Educational level**					
n (%)	Secondary	University degree	Postgraduate	No formal	Prefer not to say
Study 1	172 (34%)	221 (44.2%)	101 (20%)	4 (0.8%)	2 (0.4%)
Study 2	277(39%)	295(42%)	123 (17%)	8 (1.1%)	2 (0.3%)
**Current work status**					
	Full time	Part time	Self employed	Student	Retired
Study 1	183 (37%)	62 (12.4)	66 (13%)	28 (5.6%)	65(13%)
Study 2	260(37%)	75(11%)	74 (10.5%)	64 (9%)	97 (14%)
	Home keeper/not working or seeking	On furlough	Prefer not to say		
Study 1	53 (21%)	39 (8%)	4 (0.8%)		
Study 2	64 (9%)	54 (8%)	17 (2%)		
**Urban setting**					
	Suburban setting/ small town	Urban setting	Village or dispersed setting	Prefer not to say	
Study 1	241(48%)	165 (33%)	91 (18%)	1 (0.2%)	
Study 2	336 (48%)	244 (35%)	124 (18%)	1 (0.001%)	

### Additional measures

#### Childhood Traumatic Questionnaire [25]

A 28-item self-report questionnaire validated for clinical and non-clinical populations, which has evinced high internal reliability and good construct validity [25]. Individuals are asked to indicate on a 5-point Likert scale whether and how often they experienced emotional, physical or sexual abuse and emotional or physical neglect in their childhood. In Study 1, Cronbach’s α for each subscale was = .81, 89, 95, 95 and .71, respectively. In Study 2 Cronbach’s α = .90, .71, .84, .89 and .95.

Across both studies, 22 participants (2%) had one missing item which was replaced by the subscale mean response items. Four (0.1%) participants had two missing items which were replaced by the subscale mean response items. One participant in Study 2 skipped more than four items and as such was excluded from analysis of this measure.

#### The Brief Symptom Inventory (BSI; [26])

A 53-item self-report scale, to evaluate psychological distress and symptoms of psychiatric disorders. This scale has good internal consistency reliability and strong convergent validity [27]. Participants are asked to rate on a 5-point Likert scale whether and how much they were distressed by the different symptoms over the last week. In this study, we focused on the global severity index (GSI; Cronbach’s α in the present study was = .97). In Study 1, 22 participants (5%), skipped one item, one participant skipped two items and one participant skipped eight items. As the GSI is calculated by dividing the total number of items to which the individual responded, missing items were not replaced. In Study 2, we used the shorter 18-items version of Brief Symptom Inventory. Nine participants (1%) skipped one item in the Brief Symptom Inventory (BSI-18; [28]) (Cronbach’s α in Study 2 = .94).

#### The Reflective Function Questionnaire (RFQ) [29]

This is an 8-item scale to measure mentalizing abilities by the degree of certainty and uncertainty with which individuals utilize mental state information to understand their own and others’ behavior. The uncertainty about mental states subscale (RFQ-u) captures poor use of mental state information and a stance characterized by a lack of knowledge about mental states. The certainty about mental states subscale (RFQ-c) captures better use of mental state information and adaptive levels of certainty about mental states (Study 1 Cronbach’s α = .79, .80, respectively and for Study 2, Cronbach’s α = certainty = .83, uncertainty = .78). Across both studies, eleven participants (1%) skipped one item, and these items were replaced by the subscale mean. As with most non-clinical samples, the correlation between RFQ-c and RFQ-u was expected to be high (*r* = .70 for Study 1 and .77 for Study 2), and therefore we focused our analysis on the RFQ-u subscale only.

#### Experiences in Close Relationships Scale-Revised (ECR-R) [30]

The ECR is a 36-item measure of adult attachment style. The scale comprises two 18-item subscales measuring two attachment constructs: *attachment-anxiety* and *attachment-avoidance*, which tends to have excellent internal consistency reliability [31]. Items are rated on a 7-point Likert scale. The ECR was only applied to Study 1. Thirteen participants (2.6%) skipped one item, and these items were replaced by the subscale mean. One participant skipped ten items (27%), and as such excluded from the analysis of this measure (Cronbach’s α = avoidance = .95 and anxiety = .93).

#### General self-efficacy [32]

A 10-item self-report scale using a 4-point Likert scale to assess a general belief that one can perform novel or difficult tasks, or cope with adversity in various domains of human functioning. This scale, which was only used in Study 1, had good internal reliability (Cronbach’s α = .90).

### Statistical analysis

Study 1 sample (N = 500) was randomly split into two datasets of equal size, a “discovery sample” (n = 250) and a “confirmation sample” (n = 250). The first sample was used to investigate the factor structure of the ETMCQ using exploratory factor analysis (EFA). Item responses were treated as ordinal categorical variables, and polychoric correlations were estimated. Identification of the potential number of factors was informed by the eigenvalue >1.0 rule [33]. To confirm the model identified from the EFA, the confirmation sample was used to run a confirmatory factor analysis (CFA), with the correlation between identified factors allowed. The CFA compared a fully unconstrained model (Model 1); a 3-factor model with all 18 original items (Model 2), a 3-factor model with 15 items (i.e., excluding the item with the lowest loading on each factor) (Model 3), a 3-factor model with 15 items which also correlated residuals between items which were considered to be similar in content (Model 4). We report model fit indices for each Model (Table 3). In order to evaluate the goodness of fit of the factor structure, the following fit indices were used: the root mean square error of approximation (RMSEA), the comparative fit index (CFI), the Tucker-Lewis Index (TLI) and Standardized Root Mean Square Residual (SRMR). CFI and TFI scores above 0.9 were considered acceptable and over 0.95 considered good, whereas RMSEA and SRMR values below 0.08 and 0.05 were considered acceptable and good respectively [34, 35]. The best fitting CFA model was also constructed using the Study 2 sample. Stata16 [36] was used to conduct the EFA, and CFA was conducted using Mplus8 [37].

Correlational analyses of ETMCQ with developmental, psychological and psychopathology measures, whilst controlling for demographic features (e.g., age, gender, annual income and level of education) were conducted. Results were corrected for multiple comparisons using a 5% false discovery rate (FDR), based on the sequential Benjamini–Hochberg FDR correction algorithm [38].

The potential role of the three ETMCQ factors as mediators of the association between childhood adversity (measured by the CTQ total score) and current mental health symptoms (measured by the BSI total score) was explored using mediation analysis with the indirect effect of adversity through each of the factors estimated in a single model. Age, gender, income and level of education were included as covariates. Bias-corrected bootstrapped confidence intervals were estimated (5000 bootstrap replications). Analyses were performed separately in Studies 1 and 2, and results compared. Likewise, the moderating role of the three epistemic factors in the relationship between adversity, insecure attachment, or low self-efficacy and psychopathology was explored in both samples.

Further analyses, which were not preregistered, were then conducted to explore differences in scores on the ETMCQ between participants based on scores of their attachment style. In order to explore differences in epistemic stance based on attachment styles, participants were grouped into ‘high’ and ‘low’ scorers based on a median split of the data on both the ‘anxious’ and ‘avoidant’ subscales of the ECR-R [39]. Only Study 1 was used for this analysis as the ECR-R was not available in Study 2. Four groups were created from these splits: “Secure” (low anxious and low avoidant), “preoccupied” (high anxious, low avoidant), “dismissing” (low anxious, high avoidant) and “fearful” (high anxious, high avoidant) [40]. One-way ANOVAs and pairwise comparisons were performed between these groups for each of the three factors in Stata16. Results were corrected for multiple comparisons using a 5% false discovery rate (FDR) [38].

To aid interpretation and comparison, and as results in both studies were very similar, we report the results for both studies together.

## Results

### Demographic data

Within our sample, 27% of participants in study 1 and 18% of study 2 reported moderate to severe emotional neglect (scale score > 15) [41], 12% of each sample reported moderate to severe physical neglect (scale score >10), 11% of each sample reported moderate to severe physical abuse (scale score > 10), 18% of Study 1 and 12% of Study 2, moderate to severe emotional abuse (scale score >13), and 11% and 10% respectively reported moderate to severe sexual abuse (scale score > 8). These figures correspond with the Office for National Statistics report for the year ending March 2019, in which roughly one in five adults in England and Wales reported having experienced at least one form of abuse before the age of 16, although neglect was not recorded in the report [42].

### Factor structure of the Epistemic Trust, Mistrust and Credulity Questionnaire

#### Exploratory factor analysis

Results of the EFA supported, as expected, a three-factor model based on the eigenvalue rule (>1.0). The eigenvalues were 3.92 (one factor, proportion of total variance accounted for = 47.53%), 2.80 (two factors, 34.08%), 1.32 (three factors, 16.03%). The eigenvalue for four factors was 0.54 and as such was discounted. The factor analysis, using oblique (promax) rotation and extracting principal factors, indicated six items for each factor: (1) Trust; (2) Mistrust; (3) Credulity (loading > .30) (See Table 2). Correlations between the extracted factor scores for Trust and Mistrust (r = -.29, p < .001) and Mistrust and Credulity (r = .37, p < .001) were statistically significant, whereas Trust and Credulity were not found to correlate (r = 0.02, p = 0.70).

**Table 2:** Results of the exploratory factor analysis (Study 1; discovery sample n = 250).

	Factor loadings		
	Trust	Mistrust	Credulity
ETMCQ_2	**0.729**	0.049	0.004
ETMCQ_7	**0.688**	0.053	0.003
ETMCQ_8	**0.653**	-0.035	-0.020
ETMCQ_13	**0.676**	-0.124	0.064
ETMCQ_1	**0.592**	-0.163	0.147
ETMCQ_14	**0.458**	0.222	-0.132
ETMCQ_9	0.068	**0.706**	-0.029
ETMCQ_10	-0.029	**0.638**	0.076
ETMCQ_15	0.219	**0.631**	-0.038
ETMCQ_4	-0.123	**0.526**	0.155
ETMCQ_3	-0.327	**0.474**	-0.077
ETMCQ_16	-0.196	**0.406**	0.113
ETMCQ_5	-0.105	-0.193	**0.857**
ETMCQ_12	-0.052	0.115	**0.830**
ETMCQ_6	0.046	-0.046	**0.744**
ETMCQ_11	0.046	0.307	**0.590**
ETMCQ_17	0.033	0.325	**0.416**
ETMCQ_18	0.218	0.088	**0.413**

Note: ETMCQ = Epistemic trust, mistrust and credulity questionnaire. See S1 Table, for the content of each item.

#### Confirmatory factor analysis

**Fig 1:**
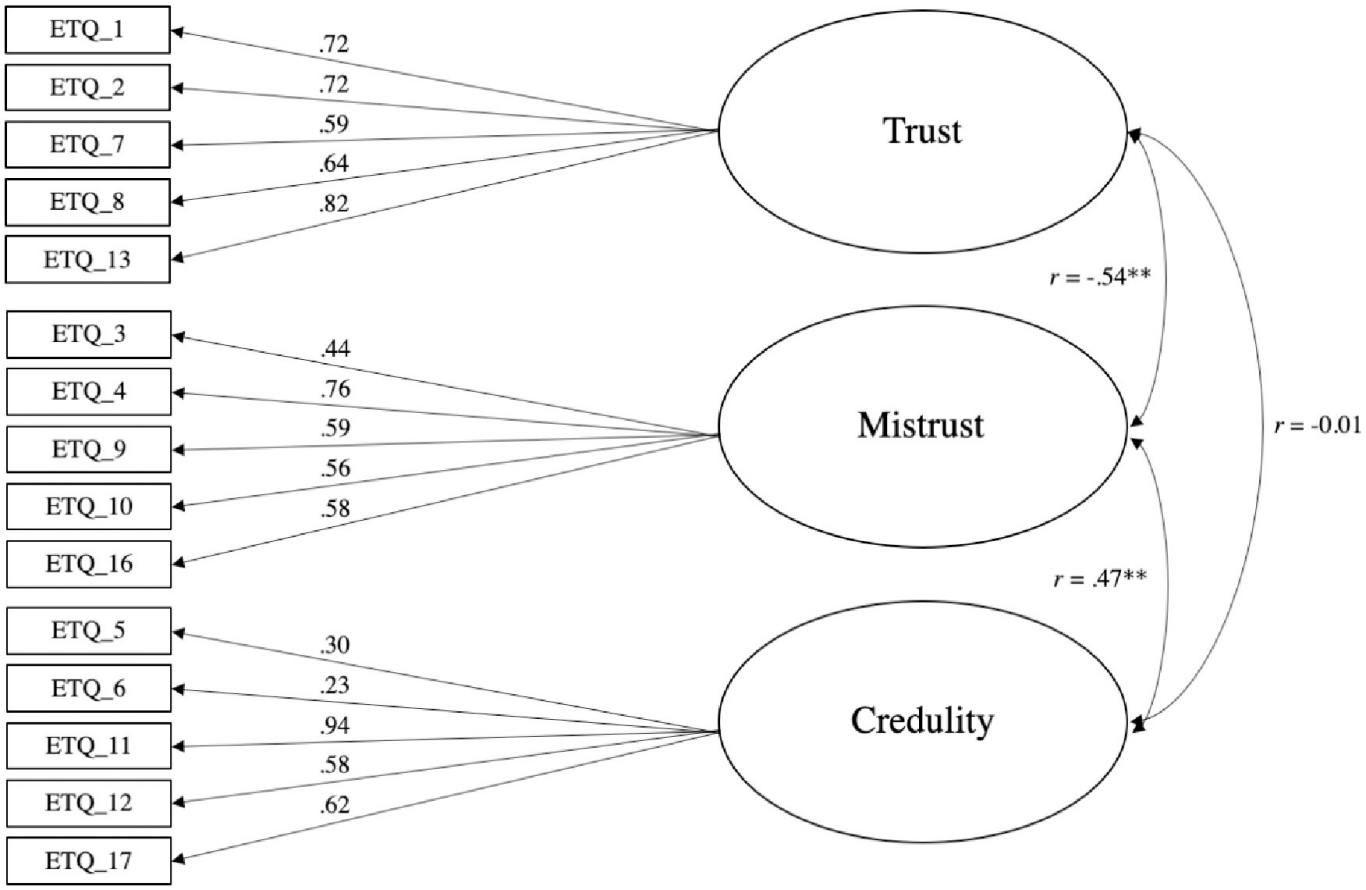
CFA with factor loadings from Study 1 (model 4, 15 items). Loadings are standardized. Rectangles indicate measured variables and circles represent latent constructs. Note the item numbering is retained from the original 18-item scale. * p < .05. ** p < .001.

Confirmatory Factor Analysis (CFA) assessed the fit of the three-factor structure using the confirmation sample from Study 1 (n = 250). The initial model (Model 1- unconstrained model—and a three-factor model with all eighteen items) did not provide a good fit to the data (see Table 3). Removing the lowest loading item on each subscale improved model fit, as did correlating the residuals between several items with similar item content and/or wording (Items 5 & 6; 7 & 8; 6 & 12; 5 & 12; 3 & 1): CFI = 0.95, TFI = 0.94, SRMR = .05 RMSEA = 0.07 (CI = 0.06–0.08) (Model 4; Table 3). The Trust and Mistrust (r = -.54, p < .001) and Mistrust and Credulity (r = .47, p< .001) factors in this model were significantly correlated, whereas Trust and Credulity were not found to correlate (r = -0.01, p = .907). All items had substantial and significant loadings in the expected direction on their respective factors (Fig 1).

**Table 3:** Results of confirmatory factor analysis (Study 1; confirmation sample n = 250).

Model	CFI	TFI	SRMR	RMSEA
1. Unidimensional	0.394	0.313	0.136	0.209 (0.2–0.22)
2. 3-factor, all items	0.849	0.825	0.075	0.105 (0.1–0.12)
3. 3-factor, 15 items	0.880	0.855	0.069	0.111 (0.10–0.12)
4. 3-factor, 15 items, CRs	0.95	0.94	0.05	0.072 (0.06–0.08)

Note: CRs = Correlated residuals; CFI = Comparative fit index; TLI = Tucker-Lewis Index; RMSEA = Root mean square error of approximation; SRMR = Standardized Root Mean Square Residual.

A similar CFA assessing the fit of the three-factor structure using the 15 items and correlating the residuals between the same five item pairs (Model 4) was performed on the Study 2 sample (n = 705). Results indicated a good model fit: CFI = 0.94, TFI = 0.92, SRMR = .05 and RMSEA = 0.08 (CI = 0.07–0.08).

#### Internal consistency

For the discovery sample of Study 1 (first half n = 250), the Cronbach’s alpha value indicated acceptable reliability for the full scale (α = 0.78), as well as for the 5-item Trust (α = 0.76), Mistrust (α = 0.72) and Credulity (α = 0.81) subscales. Analysis on the confirmation sample (n = 250) replicated findings on the first half, with acceptable internal consistency for the full scale (α = 0.75), as well as for the Trust (α = 0.81), Mistrust (α = 0.70) and Credulity (α = 0.75) subscales. Study 2 had similar acceptable consistency for the full scale (α = 0.71) and for the Credulity (α = 0.81) subscale, but lower for the Trust (α = 0.69) and Mistrust (α = 0.65) subscales.

#### Test-retest reliability

A total of 411 participants (88% out of the original sample (500)) participated in this study (199 males and 210 females; Age M = 35.33 years, SD = 24.01). Nonparametric Mann–Whitney U test for two independent groups indicated no significant differences in the ETMCQ scores between participants who participated in the retest and those who did not return. Data collection took place 3 weeks after the main questionnaire administration for Study 1. The test–retest reliability was acceptable, with Spearman *r*s = 0.73, 0.71 and 0.70 for trust, mistrust and credulity, respectively. Intraclass correlation coefficient (ICC) estimates and their 95% confident intervals were calculated based on a mean-rating (k = 411), absolute-agreement, 2-way random-effects model and indicated good reliability. Trust: ICC = .85 (95% CI (.82-.88)); Mistrust: ICC = .82 (95% CI (.77-.85)) and Credulity: ICC = .83 (95% CI (.79-.86)).

### Correlations with demographic features

The nonparametric Mann–Whitney U test for two independent groups revealed that in both studies men and women significantly differed on both Trust and Credulity subscales, with women showing higher scores than men on both subscales (Study 1: men *M* = 4.84, *SD* = 0.96; women *M* = 5.00, *SD* = 1.03, *p* = .02; η^2^ = .01; men *M* = 2.84, *SD* = 1.03; women *M* = 3.14, *SD* = 1.17, *p* = .004, η^2^ = .02 respectively; Study 2: men *M* = 4.84, *SD* = 0.88; women *M* = 5.08, *SD* = .87, *p* = .000, η^2^ = 0.03; men *M* = 2.77, *SD* = 1.02; women *M* = 3.06, *SD* = 1.17, *p* = .001, η^2^ = 0.01, respectively). In addition, in Study 2 men scored higher than women on Mistrust scale (men *M* = 4.18, *SD* = 0.88; women *M* = 4.13, *SD* = .89, *p* = .02, η^2^ = 0.006).

When examining the Spearman correlation coefficients between ETMCQ subscales and age, education and annual income in Study 1, small negative but significant negative correlations were found between age and trust (*r* = -.16, *p* = .003), between age and credulity (*r* = -.11, *p* = .04), between mistrust and annual income (*r* = -.10, *p* = .046), between credulity and annual income (*r* = -.10, *p* = .046) and between credulity and level of education (*r* = -.10, *p* = .046). No other significant correlations with demographic features were found.

Study 2 replicated the negative correlations between age and trust (*r* = -.19, *p* < .001) and between age and credulity (*r* = -.17, *p* < .001). In addition, a negative significant effect between age and Mistrust was found (*r* = -.17, *p* < .001). The negative correlation between mistrust and annual income that was found in Study 1 was only a trend in Study 2 (*r* = -.08, *p* = .07). No other effects were replicated.

### Validity of the ETMCQ

The Spearman correlation coefficients between the ETMCQ subscales and related developmental, psychological and psychopathology measures were examined while controlling for demographic features (e.g., age, gender, annual income and level of education). As expected, significant negative correlations were found between Trust and emotional and physical neglect, emotional abuse and a higher score on the global psychopathology severity index. Significant negative correlations were also found between Trust and anxious and avoidant attachment styles. In addition, as hypothesized a significant positive correlation was found between Trust and general self-efficacy (See Table 4 for Spearman’s *r*). In relation to our hypotheses on Mistrust, as expected Mistrust was positively correlated with all subtypes of neglect and abuse, with a higher score on the global psychopathology severity index, with anxious and avoidant attachment styles, and with poor mentalizing (i.e., RFQ- u). In addition, Mistrust was negatively correlated with general self-efficacy. Similar to Mistrust and as predicted, Credulity was positively correlated with all subtypes of neglect and abuse, with a higher score on the global psychopathology severity index, with anxious and avoidant attachment dimensions and with RFQ-u. In addition, Credulity was negatively correlated with general self-efficacy.

**Table 4:** Summary of Spearman intercorrelations of ETMCQ subscales, childhood trauma, mental health symptoms, mentalizing, attachment and general self-efficacy, controlling for gender, age, annual income and level of education (FDR corrected).

	Study 1 (n = 471)			Study 2 (n = 663)		
	Trust	Mistrust	Credulity	Trust	Mistrust	Credulity
1. Emotional neglect	-.31***	.32***	.18***	-.23***	.23***	.19***
2. Physical neglect	-.16**	.31***	.20***	-.08*	.13***	.23***
3. Emotional abuse	-.10*	.26***	.22***	-.12*	.25***	.22***
4. Physical abuse	-.03	.25***	.20***	-.06	.16***	.16***
5. Sexual abuse	-.03	.13*	.11*	-.03	.13*	.14***
6. RFQ-u	.01	.32***	.33***	.08	.31***	.37***
7. GSI	-.10*	.40***	.33***	-.03	.29***	.30***
8. General self-efficacy	.16**	-.15*	-.29***			
9. Avoidant-attachment	-.39***	.45***	.22***			
10. Anxious attachment	-.14**	.40***	.32***			

*Note*. FDR = False discovery rate, GSI: Global severity index

* p < 0.05

** p < 0.001

*** p < 0.0001

Study 2 Replicated all findings reported in Study 1, however correlations with subtypes of neglect and abuse were smaller (see Table 4).

### Childhood adversity, epistemic trust and mental health symptoms

**Fig 2:**
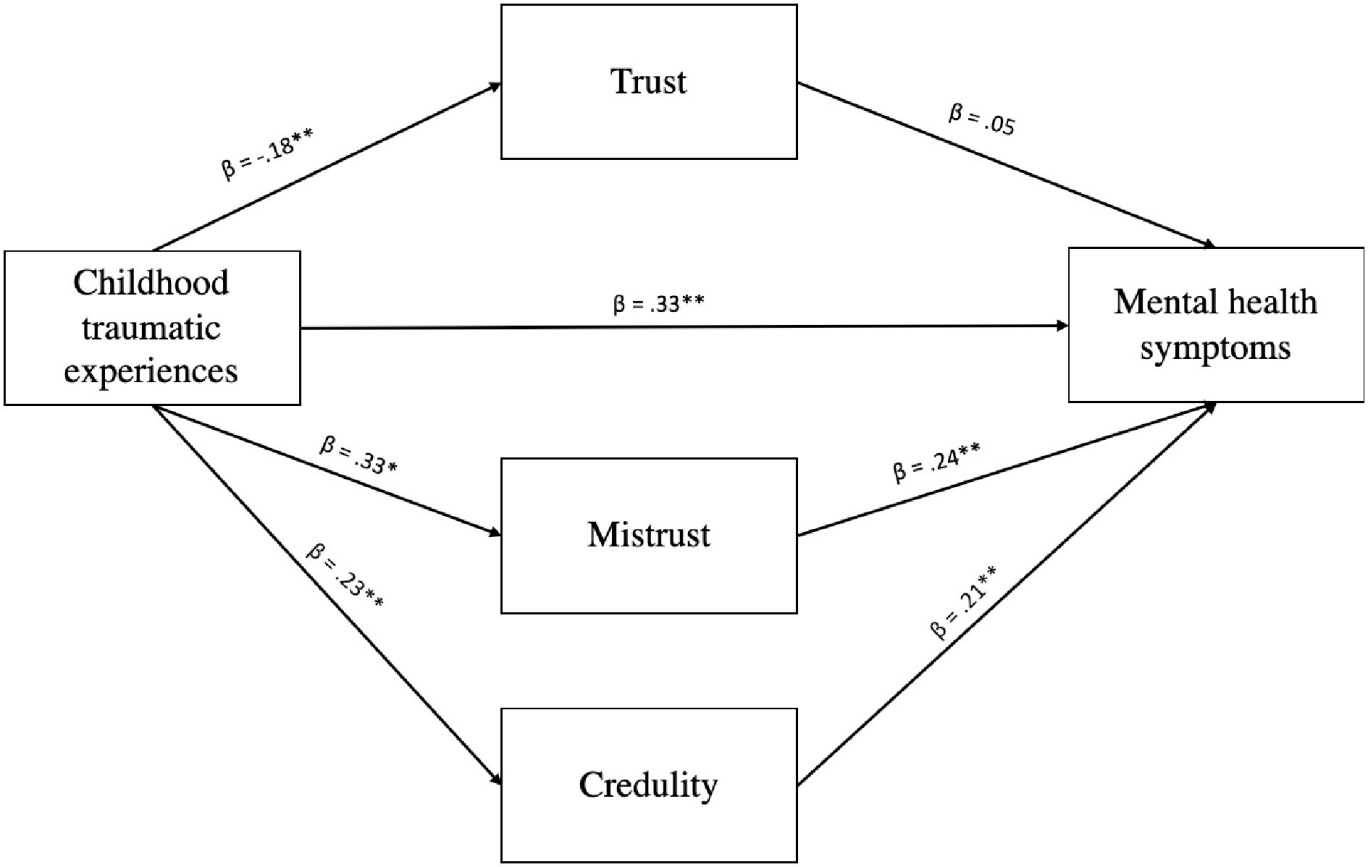
Schematic model of the mediating role of the three ETQ dimensions on the relationship between childhood trauma and mental health symptoms. * p < .05. ** p < .001.

Separate mediation models were constructed for Study 1 and Study 2, with age, gender, income and level of education included as covariates (Fig 2). The indirect effect between CTQ total score and BSI total score through the Trust factor was not significant in either study. However, the bias-corrected 95% confidence intervals (BC 95%CIs) for both the Mistrust and Credulity factors indicated the potential role of these factors as mediators of the relationship between childhood adversity and current mental health symptoms (Mistrust: Study 1—BC 95% CIs = 0.047;0.120, Study 2—BC 95%CIs = 0.001;0.055; Credulity: Study 1—BC 95% CIs = 0.024;0.081, Study 2—BC 95% CIs = 0.007;0.078). It should be noted that the magnitude of the indirect effects for Mistrust and Credulity were lower in Study 2. Our hypotheses on the moderating effects of Trust on the relationship between adversity and mental health symptoms and between low self-efficacy and insecure attachment were not supported (*p* = .14 and *p* = .41 respectively).

### Differences in epistemic stance between attachment groups

**Fig 3:**
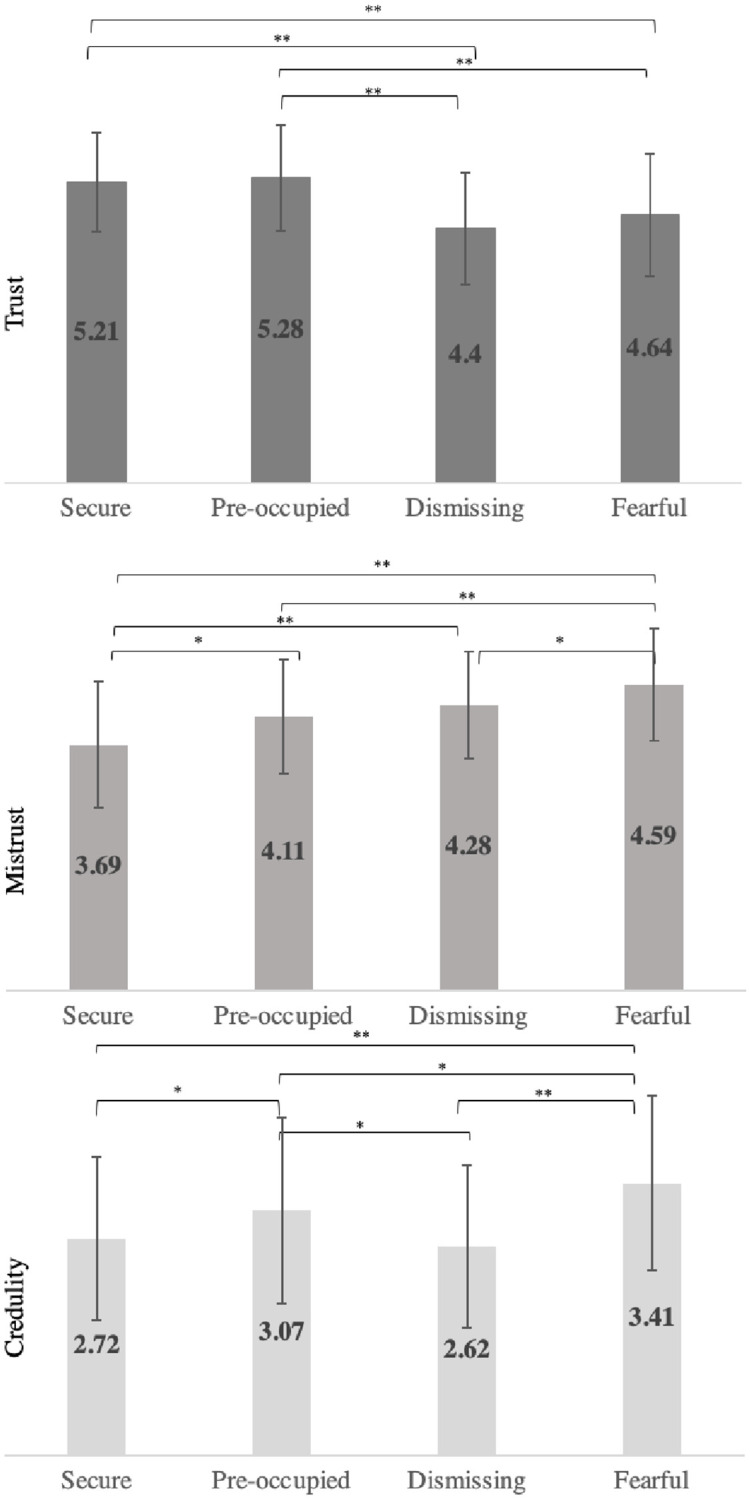
Comparison of ET subscales scores between attachment groups (p values are corrected for multiple comparisons). * p < 0.*05*. ** p < 0.*001*.

In order to further explore the relationship between different attachment styles (e.g., secure, fearful, dismissive and preoccupied) and epistemic stance, a post hoc exploration was carried out. This analysis was based on the thinking that low avoidance and anxiety (secure attachment) might be linked to high levels of Trust, while Mistrust would be most likely to characterize dismissing (low anxiety, high avoidance) individuals. The preoccupied pattern of high attachment anxiety without avoidance was expected to have similar levels of Trust to secure but might be associated with higher levels of Credulity, and the high avoidance and high anxiety which characterize attachment disorganization was expected to have the lowest levels of Trust and highest levels of Mistrust and Credulity. To investigate this post-hoc thinking, a median split of the ECR-R Anxious and Avoidant subscales (median = 3.28 Anxious; median = 2.78 Avoidant) resulted in 188 participants being coded as having “secure” attachments, 70 “preoccupied”, 63 “dismissing” and 178 “fearful” [43]. One-way ANOVAs were conducted to explore the differences in the three epistemic trust factors scores between these attachment groups (Fig 3).

In the first ANOVA, the Trust subscale was found to differ significantly between groups (F (3,495) = 20.10, p<0.001). Comparisons between groups found that the Secure and Preoccupied groups scored significantly higher than the Dismissing and Fearful groups, and that neither Secure vs Preoccupied or the Dismissing vs Fearful comparisons were statistically different.

The Mistrust subscale was also significantly different between attachment groups (F (3,495) = 32.29, *p* < 0.001). Comparisons between groups found that the mean Mistrust score was significantly lower in the securely attached group than the other three groups, and that the Fearful group’s mean was significantly higher than the other groups’. The Preoccupied and Dismissing groups’ Mistrust scores did not statistically differ.

Finally, the Credulity subscale was also found to be significantly different between groups (F (3,495) = 16.06, *p* < 0.001). The Fearful group scored higher than the other three groups, the Preoccupied scored significantly higher than the Secure group and the Dismissing group, and the Dismissing group mean was not statistically different from that of the Secure group.

## Discussion and conclusions

ET, that is, trust in communication or communicated knowledge, has traditionally been a largely theoretical concept and has only recently been applied to developmental psychopathology with direct clinical implications. Hitherto there has been little evidence that we are aware of on the relationship between ET, developmental experiences and psychopathology: the purpose of the present study was to identify initial empirical data in this domain and to create an opportunity for the further exploration of the role of this dispositional attribute in developmental psychopathology [8, 9].

In order to initiate this program of work, we in the first instance developed and tested a new questionnaire with three factors: Epistemic Trust, Mistrust and Credulity. The rationale for a three-factor model, which was confirmed in Studies 1 and 2, as opposed to a two or one factor model (trust/mistrust), derives from an understanding of the adaptiveness of epistemic vigilance in order to protect oneself from misinformation [1]. Using both exploratory and confirmatory factor analyses, the results of both studies provide initial support for the theoretically derived three-factor scale in a non-clinical representative sample, suggesting that openness to the communication of knowledge could be characterized by the three possible strategies of Trust, Mistrust and Credulity. The internal consistency and test–retest reliability and interclass correlation coefficients of the subscales were acceptable to good, suggesting coherent and relatively stable individual attributes.

The idea that these factors constitute independent factors was also supported by the correlation and mediation analyses. Clinically, our approach was informed by the presentation of individuals—often those who have experienced early adversity—whose epistemic strategy in relation to others might veer between epistemic credulity and epistemic mistrust. We have previously sought to explain the particularly damaging nature of such experiences in terms of generating epistemic petrification and/or the loss of appropriate vigilance. Our findings on the relationship between a reported history of childhood adversity and the three ETMCQ factors provide initial data in support of this position. The findings in both studies that overall neglect, but not abuse, was negatively associated with Trust, supports the idea that neglect and abuse have different developmental trajectories [44]. Humphreys and Zeanah distinguished between two different types of deviations from the expectable environment: inadequate input (neglect/deprivation) and harmful input (abuse/trauma) [45]. Neurodevelopmental models also suggest that threat (including experiences involving harm or threat of harm to a child) and deprivation, (entailing an absence of expected inputs from the environment during development, such as support, nurturance, and cognitive and social stimulation) involve different forms of neural dysfunction [46–48]. Threat-related early adversity appears to be associated with reduced amygdala, medial prefrontal cortex (mPFC) and hippocampal volume and heightened amygdala activation to threat. In contrast, children exposed to deprivation manifest reduced volume and altered function in frontoparietal areas involved in higher order cognition. Consistent with these models, we have suggested that neglect can be understood as involving an absence of exposure to the social co-construction of internal states that is necessary for creating the interpersonal understanding (epistemic match) on which the emergence of trust depends [49]. Both neglect and abuse are strongly positively associated with Mistrust and Credulity which is consistent with the assumption that adversity can generate long term disruptions in the capacity to adapt by compromising social learning [19, 20] as a result of suspicion and inability to accurately identify trustworthy sources. It is encouraging that these associations emerged in a community sample with relatively low prevalence and severity of early adversity. Further confirmation is needed from clinical samples with higher levels of early adversity.

As predicted, we found that Mistrust and Credulity scores were associated with higher scores on the global psychopathology severity index and that both factors partially mediated the link between early adversity and psychopathology. These results are consistent with our assumption that both Mistrust and Credulity may be expected to undermine adaptation and increase the developmental risk of mental health problems [50, 51]. Congruent with the finding that secure childhood attachment turns out not to be a protective factor for mental disorder [52], the Trust factor was not associated with reduced levels of mental health symptoms, nor was it a moderator in buffering against childhood adversity, so could not be regarded as a resilience factor. We may speculate that the presence of Trust may be a default mode of social functioning and in that sense a somewhat neutral value. Trusting over and above the average brings no additional benefit from a clinical standpoint. We presume that Trust may bring with it other benefits for social functioning, arising out of an enhanced capacity for acquiring new information from social communication [53], but this remains to be explored in future studies, and in clinical samples in particular. These findings on psychopathology are in line with the idea, which we have previously discussed [24], that working to reduce epistemic mistrust and credulity may be a crucial common factor in the effectiveness of psychotherapeutic interventions enabling improved adaptation to interpersonal environments. Future research might explore these issues in clinical populations and in relation to psychotherapy outcomes.

In relation to attachment styles, we found strong and positive relationships between insecure attachment and Credulity and Mistrust, and a smaller negative relationship between insecurity and Trust. Overall, these associations highlight the conceptual closeness of the domains of ET and attachment. Some philosophers consider the infant’s potential to trust unreflectively, confidently relying on a caring and responsively mentalizing relationship, as the foundation of the experience of trust [54]. Secure attachment reflects this basic trust in infancy, the confident reliance on a caring and responsively mentalizing or mind-minded relationship [55] pertaining throughout life [56]. Conversely, we can think of insecure infant strategies of avoidance of relying on others and the excessive protests of anxious-ambivalent attachment as both exemplifying early manifestations of different types of disruptions in the capacity to trust the communication of information. Avoidance is most strongly associated with Mistrust, which might be attributed to a dismissive approach towards reliance on others. Mistrust may reflect an experience of concern about the reliability or personal relevance of newly acquired knowledge characteristic of insecure attachment. Our post-hoc analysis explored these relations further and have suggested that preoccupied individuals have no or little difficulty with Trust, but tend to score highly on Credulity, suggesting an overvaluing of the communication of others at the expense of epistemic self-agency. A fearful attachment style (high avoidance and anxiety) is associated with perhaps the most acute epistemic dilemma: scoring low on Trust and high on both Mistrust and Credulity, fearful individuals are not only mistrusting but are also vulnerable to misinformation. This combination may result in epistemic petrification, which reduces flexibility, adaptation and openness to new information and thus becomes an obstacle to resilience and salutogenesis [7]. It should be noted that the results on the ETMCQ and attachment were only collected in one of the studies and require replication.

Just as attachment and epistemic stance have been conceptualized as closely related, mentalizing (the capacity to understand the mental states that underpin behavior) is also an important part of this developmental constellation [57, 58]. We have suggested that exposure to being inadequately mentalized by others creates a barrier to the transmission of culturally pertinent knowledge by impeding the development of appropriately trusting relationships [9, 59]. As hypothesized, we found Credulity and Mistrust were positively correlated with difficulties in understanding mental states as measured by the RFQ. If replicated with other samples and other measures of mentalizing, this could confirm an important aspect of recent theoretical views concerning the relationship between epistemic stance and interpersonal understanding [7].

The particular significance of the negative relationship between Credulity and general self-efficacy is suggestive of the interpersonal implications of a lack of agency. General self-efficacy (GSE) may be understood as a measure of agentiveness [60]. We based our hypothesis on the relationship between GSE and Credulity on the assumption that a mentalizing relationship by its very nature involves the recognition of agency and our emphasis on the significance of that recognition as a generic ostensive cue. Abusive or neglecting experiences, on the other hand, imply the denial or disregard of agentive selfhood [16]. We suggest that lack of agentiveness, as assessed in the GSE, may be closely associated with the social cognitive processes underpinning a credulous strategy in relation to others, as credulity tends to involve the abdication of one’s capacity to parse information or opinions in favor of someone else’s judgement. Given the role of credulity in generating vulnerability in relationships, this may be an issue of clinical significance which would benefit from further research.

On examining the relationship between the demographics and the three factors, we found certain effects that were replicated in both studies. There was a significant difference between genders, with women scoring significantly higher than men on trust and credulity and men scoring more highly on mistrust. Small negative correlations were found between age and trust which may be part of a trend of increasing suspiciousness with age [61]. Negative correlations were also found between mistrust and annual income, which may be explained by less reliance on others and greater reliance on the self amongst those with higher socio-economic status [62]. The association between credulity and level of education and annual income may be seen as reflecting the generic advantage of critical thinking that education can offer [63]. These associations, although relatively small in the current samples, highlight the potential importance of controlling for demographic features in future research.

Taken together, findings across both studies are encouraging in terms of the reliability and validity of the ETMCQ. Having a scale that provides an empirical measure of what until now has been largely a theoretical concept opens new avenues for future research.

There are also limitations to the present studies that need to be considered. The samples were community-based rather than clinical but many of the conclusions here have reached into the clinical domain. This is based on the continuum based equifinality notion within developmental psychopathology [64], but clearly further research on the relationship between the three factors in relation to explicit mental disorder diagnoses would be necessary to substantiate the suggestions in this paper. In particular, as much of the clinical inspiration for the measure and the interpretation of its association with self-rating scales in attachment and general psychopathology comes from the study of borderline personality disorder, the relationship between the ETMCQ scales and borderline personality disorder would be desirable. Second, as this study used a cross-sectional design, and although the direction of effect is based on the assumption that childhood experience refers to the past, while psychopathology and epistemic stance are current experiences, we cannot rule out alternative explanations such as selective recall of adverse events in individuals with current difficulties. Future longitudinal studies should test these directions and investigate the long-term effects of the epistemic stance on mental health symptoms and psychological constructs. Third, we did not assess general interpersonal trust [65], which would generate discriminant validity of the epistemic trust factor. The prediction from the model would suggest additional specific variance accounted for by ETMCQ when general trust was controlled for. Fourth, although we undertook a limited replication of Study 1 in Study 2, we are acutely aware of the problems of replication within psychology and the unproductive lines of inquiry isolated findings can create in the field [66]. Further studies would therefore beneficially seek to replicate ours. Finally, our findings focused only on self-report measures: future studies assessing the association between the ETMCQ and experimental designs which test laboratory social learning. We assume that the real time learning and memory performance of those scoring high on the ETMCQ would be impaired in tasks where learning involved detection of personal relevance and the need to reflect a mental state of the sender.

To conclude, and to the best of our knowledge, this is the first attempt to quantify a three-factor model of epistemic trust, mistrust and credulity as an individual attribute and explore its associations with childhood trauma, attachment, mentalizing, and mental health symptoms.

## Supporting information

S1 Table Items in the Epistemic Trust, Mistrust and Credulity Questionnaire. (DOCX)

S1 Study (CSV)

S2 Study (CSV)
