# Supplementary material for: Development and validation of a self-report measure of epistemic trust
Source: PLoS One. 2021 Apr 16;16(4):e0250264. doi: 10.1371/journal.pone.0250264 (PMC8051785; doi:10.1371/journal.pone.0250264)
Supplement: S1 Table — (DOCX) [file pone.0250264.s001.docx]

**S1 Table. Items in the Epistemic Trust, Mistrust and Credulity Questionnaire**

| Item number  (15-item scale) | Item’s original number (18-item scale) | Item | Subscale |
| --- | --- | --- | --- |
| 1 | 1 | I usually ask people for advice when I have a personal problem. | Trust |
| 2 | 2 | I find information easier to trust and absorb when it comes from someone who knows me well. | Trust |
| 3 | 3 | I’d prefer to find things out for myself on the internet rather than asking people for information. | Mistrust |
| 4 | 4 | I often feel that people do not understand what I want and need. | Mistrust |
| 5 | 5 | I am often considered naïve because I believe almost anything that people tell me. | Credulity |
| 6 | 6 | When I speak to different people, I find myself easily persuaded by what they say even if this is different from what I believed before. | Credulity |
| 7 | 7 | Sometimes, having a conversation with people who have known me for a long time helps me develop new perspectives about myself. | Trust |
| 8 | 8 | I find it very useful to learn from what people tell me about their experiences. | Trust |
| 9 | 9 | If you put too much faith in what people tell you, you are likely to get hurt. | Mistrust |
| 10 | 10 | When someone tells me something, my immediate reaction is to wonder why they are telling me this. | Mistrust |
| 11 | 11 | I have too often taken advice from the wrong people. | Credulity |
| 12 | 12 | People have told me that I am too easily influenced by others | Credulity |
| 13 | 13 | If I don’t know what to do, my first instinct is to ask someone whose opinion I value. | Trust |
| 14 | 16 | I don’t usually act on advice that I get from others even when I think it’s probably sound. | Mistrust |
| 15 | 17 | In the past, I have misjudged who to believe and been taken advantage of. | Credulity |
